# Health-related quality of life, health literacy and COVID-19-related worries of 16- to 17-year-old adolescents and parents one year into the pandemic: a cross-sectional study

**DOI:** 10.1186/s12889-022-13737-1

**Published:** 2022-07-09

**Authors:** Hilde Timenes Mikkelsen, Siv Skarstein, Sølvi Helseth, Milada Cvancarova Småstuen, Kristin Haraldstad, Gudrun Rohde

**Affiliations:** 1 Department of Health and Nursing, Faculty of Health and Sport Sciences, University of Agder, Postbox 422, 4604 Kristiansand, Norway; 2 Department of Nursing and Health Promotion, Faculty of Health Sciences, Oslo Metropolitan University, Oslo, Norway; 3 Department of Clinical Research, Sorlandet Hospital, Kristiansand, Norway

**Keywords:** health-related quality of life, health literacy, COVID-19, worries, adolescents, parents

## Abstract

**Background:**

The uncertain and challenging situation caused by the COVID-19 pandemic affects adolescents and their parents in an exceptional way. More knowledge of health-related quality of life (HRQoL), health literacy (HL) and COVID-19-related worries in adolescents and parents 1 year into the pandemic is needed. The present study aimed to describe HRQoL, HL and COVID-19-related worries of 16- to 17-year-old adolescents and parents of adolescents. Further, to assess the strength of associations between gender, HL, COVID-19-related worries and HRQoL.

**Methods:**

A cross-sectional study involving 215 adolescents and 320 parents was conducted, exploring HRQoL, HL, COVID-19-related worries and sociodemographic variables. KIDSCREEN-10 and RAND-36 were used to measure HRQoL. Data were analyzed using bivariate methods, multiple linear regression and robust regression.

**Results:**

Adolescents’ HRQoL was notably lower compared to previous Norwegian studies and European norms. Parents’ HRQoL was comparable to Norwegian norms. Adolescents and parents reported moderate-to-high HL and high degrees of COVID-19-related worries. Females reported significantly lower HRQoL and more worries compared to males. In adolescents, higher HL was significantly associated with higher HRQoL. COVID-19-related worries were not significantly associated with HRQoL. In parents, higher HL in the “understand health information” domain was significantly associated with higher HRQoL for mental well-being (mental component sum scores [MCS]) and with lower HRQoL for physical well-being (physical component sum scores [PCS]). Being worried a lot about infecting others and about family/friends becoming sick was significantly associated with higher MCS and lower MCS, respectively. COVID-19-related worries were not significantly associated with PCS.

**Conclusions:**

Our results indicate that the pandemic has a major negative impact on adolescents’ HRQoL. Parents’ HRQoL remained unchanged and comparable to previous studies. Our study demonstrates that HL, gender and COVID-19-related worries are significantly associated with adolescents’ and parents’ HRQoL, indicating that efforts aimed at increasing their HL might indirectly affect their HRQoL as well and that gender-specific interventions or strategies could be beneficial.

**Supplementary Information:**

The online version contains supplementary material available at 10.1186/s12889-022-13737-1.

## Background

In 2020, coronavirus disease 2019 (COVID-19) spread around the world, and in March 2020, the World Health Organization (WHO) characterized the situation as a pandemic [1]. The pandemic led to major changes in people’s lives through protective strategies aimed at limiting the transmission of COVID-19—for example, the practice of hand hygiene, the use of face masks in public places, social distancing instructions, lockdowns, closed or limited leisure activities, homeschooling and increased use of home offices. One year later, despite an overall encouraging picture related to COVID-19 vaccines, mutations of the virus pose additional challenges, and the pandemic still affects daily life.

The uncertain situation caused by the pandemic affects adolescents and their families in an exceptional way [2–5]. The negative effects of social distancing might be particularly challenging for adolescents, for whom peer interaction is especially important [6, 7]. Studies conducted during the pandemic have revealed increased loneliness in both adolescents and adults [3, 8, 9]. COVID-19-related restrictions and potential health risks affect emotions and perceived stress in adolescents and parents and may be associated with COVID-19-related worries and deteriorated mental health [3, 9–16]. More perceived stress and deteriorated mental health have been identified in parents as compared to adults without children in the same household [3, 10]. Further, the pandemic has major economic implications for several families [5, 10, 13]. During the pandemic, more frequent negative and positive interactions between parents and adolescents have been reported—for example, increased conflicts, more quality time with family and increased feelings of closeness [2, 10, 17–19]. The pandemic has affected many lives both positively and negatively [14, 15, 19].

The pandemic has been linked to lower life satisfaction and reduced health-related quality of life (HRQoL) in adolescents and adults [4, 16, 20–23]. HRQoL is a multidimensional construct that includes the individual’s subjective perspectives on the physical, psychological, functional and social aspects of health [24]. Previous studies have demonstrated that sociodemographic factors (e.g., male gender and higher socioeconomic status [SES]) are associated with higher HRQoL in adolescents and adults both before and during the initial phase of the pandemic [4, 16, 21, 25–27]. However, more knowledge of HRQoL in adolescents and parents 1 year into the COVID-19 pandemic is needed.

For the protective strategies to be successful, COVID-19-related information and advice need to be understood and acted upon by the population. Hence, individuals’ health literacy (HL) can have an important impact on the effective use of health knowledge [21]. HL is a concept within health promotion that represents the skills that determine the ability and motivation of individuals to gain access to, interpret, understand and use health information to maintain and promote good health [28].

A relationship between HL and health behaviors in adolescents has been revealed [29]. Thus, more knowledge on adolescents’ HL during the pandemic is needed, as they are increasingly becoming independent and responsible for their health behaviors [21]. Although adolescents are less likely to become seriously ill from COVID-19 [30], their willingness to, for example, follow social distancing guidelines is essential for reducing the risk of spreading the virus. Further, parents are important role models; thus, more knowledge of HL in parents of adolescents during the pandemic is needed.

Low HL has been associated with reduced quality of life [31, 32]. A recent study among Norwegian adolescents during the pandemic found that HL is positively associated with HRQoL [8]. HL has also been found to be a protective factor for improving adults’ HRQoL during the pandemic [26, 33]. However, the literature regarding the impact of HL on adolescents’ and parents’ HRQoL during the pandemic is scarce. Increased knowledge of this association will be valuable for health promotion interventions and policy.

The aim of this study is to describe HRQoL of 16- to 17-year-old adolescents and parents of adolescents, their HL and degree of COVID-19-related worries about 1 year into the pandemic, and to assess the strength of associations between gender, HL, COVID-19-related worries and HRQoL.

## Methods

### Sample and data collection

This study is a part of *Start Young – Quality of Life and Pain in Generations* [34]—a Norwegian longitudinal study aiming to acquire new knowledge about HRQoL and pain in adolescents and their parents. The present study uses data collected from January to February 2021, around 2 years after the overall study’s baseline data collection and approximately 1 year into the COVID-19 pandemic.

Potential participants of this study were 647 adolescents and 561 adults (all parents of a 16- to 17-year-old adolescent) who had participated in the baseline study and thereby provided their telephone numbers. The potential participants received a text message with an invitation to participate in the study and a safe link to the questionnaire. Informed consent was given at the beginning of the questionnaire. In total, 215 adolescents (response rate: 33.2%) and 320 parents (response rate: 57.0%) took part in this study.

Data collection was carried out through a web-based questionnaire the participants completed in their spare time. We used a safe data server to store the collected data [35]. All study procedures were approved by an ethics committee at the University of Agder and by the Norwegian Centre for Research Data (Ref:60981).

### Measures

An electronic survey tool that consecutively administered the following questionnaires was used. Most questions included a neutral option, resulting in all items being answered. All questionnaires using sum scales showed satisfactory Cronbach’s alpha values above 0.7 (see Additional file 1).

The first part of the questionnaire included self-reported data on sociodemographic variables. Adolescents answered questions about gender, age, parents’ birthplace, adult members of the household and parental work status. Parents answered questions about age, gender, marital status, education level, work status and household income.

*HRQoL in adolescents* was assessed using the KIDSCREEN-10 Index [36, 37]—a unidimensional self-report measure of HRQoL that represents a global score for the dimensions of the longer KIDSCREEN versions [38]. KIDSCREEN-10 consists of 10 items covering perceptions of physical well-being, psychological well-being, autonomy and parent relations, social support and peers, and school environment. We computed Rasch scores and transformed them into t-values in line with the KIDSCREEN handbook [38]. These t-values are normed to a mean (standard deviation [SD]) of 50 (10) and can be used to make comparisons with international t-values. The Norwegian KIDSCREEN-10 is considered valid and reliable [39].

*HRQoL in parents* was assessed using the 36-item Medical Outcomes Study Short Form (RAND-36). RAND-36 is a generic questionnaire consisting of eight domains that can be combined into a physical component sum score (PCS), reflecting physical health (general health, bodily pain, physical function and role limitations), and a mental component sum score (MCS), reflecting mental health (mental health, vitality, social function and role limitations) [40, 41]. The Norwegian RAND-36 is considered valid and reliable [42].

*HL in adolescents* was assessed using the 10-item Health Literacy in School-Aged Children (HLSAC) questionnaire [43], which includes two items from each of the following theoretical components: theoretical knowledge, practical knowledge, critical thinking, self-awareness and citizenship. Based on the sum score, HL levels can be defined as follows: “low” (score 10–25), “moderate” (score 26–35) or “high” (score 36–40) [44, 45]. The Norwegian HLSAC has been used among adolescents and has shown satisfactory internal consistency and a dominant first factor with eigenvalue = 3.88 [21].

*HL in parents* was assessed using the Health Literacy Questionnaire (HLQ)—a generic, multidimensional instrument [46] comprising 44 questions representing nine independent HL domains. We used five of the nine HLQ-domains that were considered the most relevant for our purpose. The chosen domains focus on having sufficient information to manage health, actively managing health, and understanding health information, as well as on the appraisal of health information and the ability to find good health information. Each domain comprises four to six items. The domain scores are calculated as the average of the item scores. Higher scores indicate better HL. The Norwegian HLQ is considered reliable and valid [47].

*COVID-19-related-worries in adolescents and parents* were assessed using selected questions derived from the Norwegian study *Adolescents in Oslo in the Time of the COVID-19 Pandemic* [14, 48]. We used two questions concerning whether the COVID-19 pandemic had changed the participants’ lives positively and/or negatively. We used six questions concerning different COVID-19-related worries: becoming sick and infecting others, as well as worried about family/friends becoming sick, school grades (for adolescents) or work (for parents), the family’s economy and Norwegian economy. In the regression analyses, we selected “worried about infecting others with COVID-19” and “worried about family/friends becoming sick” as independent variables because these were the COVID-19-related worries most highly reported by adolescents and parents.

### Data analyses

Descriptive statistics were calculated for all variables stratified by gender and presented as counts and percentages for categorical variables and as means with SDs or medians with min/max for continuous variables, as appropriate. Crude associations between pairs of variables were assessed using the chi-square test for categorical variables and an independent samples t-test or Mann–Whitney U test for continuous variables. Some variables with several categories were recoded into fewer categories to fulfill the assumptions for validity of the chi-square test [49]. For variables where these assumptions were not met, associations between pairs of variables were not assessed. This is explained in the footnotes of Tables 1 and 2.

**Table 1:** Sociodemographic characteristics of adolescents and parents

Adolescent characteristics	Total (*N* = 215)	Boys (*n* = 66)	Girls (*n* = 149)	*P*-value
Age, median (min, max)	16.0 (16.0, 18.0)	16.0 (16.0, 18.0)	16.0 (16.0, 18.0)	.942
Adult members of the household, N (%) ^a^				.927
Both parents	154 (71.6)	48 (72.7)	106 (71.1)	
Alternates between two parents	32 (14.9)	10 (15.2)	22 (14.8)	
One parent and/or other caregivers	29 (13.5)	8 (12.1)	21 (14.1)	
**Parent characteristics**	Total (*N* = 320)	Men (*n* = 62)	Women (*n* = 258)	*P*-value
Age, mean (SD)	47.6 (4.6)	49.1 (4.8)	47.2 (4.5)	**.022**
Marital status, N (%) ^b^				.528
Married/cohabitant	252 (78.8)	47 (75.8)	205 (79.5)	
Single/divorced	68 (21.3)	15 (24.2)	53 (20.5)	
Education level, N (%) ^c^				.558
≤ 12 years and/or certificate of apprenticeship	61 (19.1)	9 (14.5)	52 (20.2)	
13–15 years (< 4 years of higher education)	73 (22.8)	16 (25.8)	57 (22.1)	
≥ 16 years (≥ 4 years of higher education)	186 (58.1)	37 (59.7)	149 (57.8)	
Work status, N (%) ^d^				
Yes, full time	250 (78.1)	59 (95.2)	191 (74.0)	
Yes, part time	42 (13.1)	1 (1.6)	41 (15.9)	
No, not employed	28 (8.8)	2 (3.2)	26 (10.1)	
Household income, N (%) ^d, e^				
≤ 450,000 NOK/year	19 (5.9)	1 (1.6)	18 (7.0)	
451,000–750,000 NOK/year	50 (15.6)	8 (12.9)	42 (16.3)	
751,000–1,000,000 NOK/year	63 (19.7)	5 (8.1)	58 (22.5)	
> 1,000,000 NOK/year	188 (58.8)	48 (77.4)	140 (54.3)	

Continuous variables analyzed using an independent t-test and Mann–Whitney U test. Categorical variables analyzed with χ^2^-test

*P*-values marked with bold print indicate statistically significant differences between gender (P ≤ 0.05)

*SD* Standard deviation

^a^The variable was recoded into three categories: “Both parents,” “Alternates between two parents” and “One parent and/or other caregivers” (one parent and one step-parent, one parent, other caregivers)

^b^The variable was dichotomized as “Married/cohabitant” or “Single/divorced” (single, divorced, widowed)

^c^The variable was recoded into three categories: “≤ 12 years and/or certificate of apprenticeship” (9 years, 10–11 years, 12 years, certificate of apprenticeship), “13–15 years (< 4 years of higher education)” and “≥ 16 years (≥ 4 years of higher education)”

^d^Assumptions for chi-square analysis were not fulfilled. Associations between pairs of variables were not assessed

^e^The variable was recoded into four categories: “≤ 450,000 NOK/year” (< 250,000 NOK/year and 250,000–450,000 NOK/year), “451,000–750,000 NOK/year,” “751,000–1,000,000 NOK/year” and “> 1,000,000 NOK/year”

**Table 2:** Descriptive data for health-related quality of life, health literacy and COVID-19-related worries of adolescents and parents

Adolescent characteristics	Total (*N* = 215)	Boys (*n* = 66)	Girls (*n* = 149)	*P*-value
HRQoL, mean (SD) ^a^	44.3 (7.8)	47.5 (8.8)	42.8 (6.8)	**<.001**
Health literacy, median (min, max) ^b^	34 (20, 40)	35 (20,40)	34 (21,40)	.096
The COVID-19 pandemic changing life negatively, N (%) ^c^				.092
No, not at all	29 (13.5)	12 (18.2)	17 (11.4)	
Yes, a little	78 (36.3)	28 (42.4)	50 (33.6)	
Yes, considerably	108 (50.2)	26 (39.4)	82 (55.0)	
The COVID-19 pandemic changing life positively, N (%) ^c^				**.047**
No, not at all	42 (19.5)	19 (28.8)	23 (15.4)	
Yes, a little	115 (53.5)	34 (51.5)	81 (54.4)	
Yes, considerably	58 (27.0)	13 (19.7)	45 (30.2)	
Worried about becoming sick with COVID-19 ^d^				**.032**
Not worried at all	92 (42.8)	37 (56.1)	55 (36.9)	
A little worried	99 (46.0)	23 (34.8)	76 (51.0)	
Worried a lot	24 (11.2)	6 (9.1)	18 (12.1)	
Worried about infecting others with COVID-19 ^d^				**.001**
Not worried at all	15 (7.0)	9 (13.6)	6 (4.0)	
A little worried	58 (27.0)	25 (37.9)	33 (22.1)	
Worried a lot	142 (66.0)	32 (48.5)	110 (73.8)	
Worried about family/friends becoming sick with COVID-19 ^e^				**.021**
Not worried at all	17 (7.9)	9 (13.6)	8 (7.9)	
A little worried	67 (31.2)	25 (37.9)	67 (31.2)	
Worried a lot	131 (60.9)	32 (48.5)	131 (60.9)	
Worried about my school grades ^e^				.120
Not worried at all	72 (33.5)	26 (39.4)	46 (30.9)	
A little worried	84 (39.1)	28 (42.4)	56 (37.6)	
Worried a lot	59 (27.4)	12 (18.2)	47 (31.5)	
Worried about my family’s economy ^d^				.503
Not worried at all	152 (70.7)	49 (74.2)	103 (69.1)	
A little worried	32 (14.9)	7 (10.6)	25 (16.8)	
Worried a lot	31 (14.4)	10 (15.2)	21 (14.1)	
Worried about the economy in Norway ^d^				**.001**
Not worried at all	103 (47.9)	42 (63.6)	61 (40.9)	
A little worried	88 (40.9)	15 (22.7)	73 (49.0)	
Worried a lot	24 (11.2)	9 (13.6)	15 (10.1)	
**Parent characteristics**	Total (*N* = 320)	Men (*n* = 62)	Women (*n* = 258)	*P*-value
HRQoL				
RAND-36 PCS, median (min, max) ^e^	54.7 (14.7, 67.5)	55.0 (35.2, 62.4)	54.6 (14.7, 67.5)	.152
RAND-36 MCS, median (min, max) ^e^	54.4 (10.1, 67.3)	55.6 (28.5, 65.8)	54.0 (10.1, 67.2)	**.041**
Health literacy ^f^				
Having sufficient information to manage my health, mean (SD)^g^	3.2 (0.5)	3.3 (0.5)	3.2 (0.4)	.480
Actively managing my health, mean (SD) ^g^	3.0 (0.5)	2.9 (0.5)	3.0 (0.5)	.117
Appraisal of health information, mean (SD) ^g^	2.9 (0.5)	2.8 (0.4)	2.9 (0.5)	.954
Ability to find good health information, mean (SD) ^h^	4.9 (0.5)	4.1 (0.5)	4.0 (0.5)	.168
Understand health information well enough to know what to do, mean (SD) ^h^	4.1 (0.5)	4.1 (0.5)	4.1 (0.5)	.324
The COVID-19 pandemic changing life negatively, N (%) ^c^				.539
No, not at all	62 (19.4)	9 (14.5)	53 (16.6)	
Yes, a little	155 (48.4)	31 (50.0)	124 (48.1)	
Yes, considerably	103 (32.2)	22 (35.5)	81 (31.4)	
The COVID-19 pandemic changing life positively, N (%) ^c^				**<.001**
No, not at all	103 (32.2)	32 (51.6)	71 (27.5)	
Yes, a little	165 (51.6)	27 (43.5)	138 (53.5)	
Yes, considerably	52 (16.3)	3 (4.8)	49 (19.0)	
Worried about becoming sick with COVID-19 ^d^				**.013**
Not worried at all	161 (50.3)	41 (66.1)	120 (46.5)	
A little worried	139 (43.4)	20 (32.3)	119 (46.1)	
Worried a lot	20 (6.3)	1 (1.6)	19 (7.4)	
Worried about infecting others with COVID-19^d^				**.003**
Not worried at all	58 (18.1)	19 (30.6)	39 (15.1)	
A little worried	142 (44.4)	29 (46.8)	113 (43.8)	
Worried a lot	120 (37.5)	14 (22.6)	106 (41.1)	
Worried about family/friends becoming sick with COVID-19 ^d^				**.005**
Not worried at all	44 (13.8)	16 (25.8)	28 (10.9)	
A little worried	151 (47.2)	29 (46.8)	122 (47.3)	
Worried a lot	125 (39.1)	17 (27.4)	108 (41.9)	
Worried about work ^d^				.729
Not worried at all	225 (70.3)	42 (67.7)	183 (70.9)	
A little worried	66 (20.6)	15 (24.2)	51 (19.8)	
Worried a lot	29 (9.1)	5 (8.1)	24 (9.3)	
Worried about my family’s economy ^d^				.989
Not worried at all	224 (70.0)	43 (69.4)	181 (70.2)	
A little worried	70 (21.9)	14 (22.6)	56 (21.7)	
Worried a lot	26 (8.1)	5 (8.1)	21 (8.1)	
Worried about the economy in Norway ^d^				.891
Not worried at all	78 (24.7)	16 (25.8)	63 (24.4)	
A little worried	172 (53.8)	34 (54.8)	138 (53.5)	
Worried a lot	69 (21.6)	12 (19.4)	57 (22.1)	

Continuous variables analyzed using an independent t-test and Mann–Whitney U test. Categorical variables analyzed with χ^2^-test

*P*-values marked with bold print indicate statistically significant differences between gender (*P* ≤ 0.05)

HRQoL, health-related quality of life; SD, standard deviation; PCS, physical component sum score; MCS, mental component sum score

^a^KIDSCREEN-10. Rasch scores were computed and transformed into t-values, with a mean of 50 and an SD of 10. Higher values indicate higher levels of HRQoL

^b^Sum score obtained from the Health Literacy in School-Aged Children questionnaire (min–max: 10–40). Higher scores indicate higher levels of health literacy

^c^The variable was recoded into three categories: “No, not at all,” “Yes, a little” and “Yes, considerably” (yes, partly, a lot, considerably)

^d^The variable was recoded into three categories: “Not worried at all,” “A little worried” and “Worried a lot” (quite worried, worried a lot)

^e^RAND-36 scores range from 0 to 100, where 100 means perfect health

^f^Sum scores obtained from the Health Literacy Questionnaire. Higher scores indicate higher levels of health literacy

^g^Scales with a possible total score of 1–4

^h^Scales with a possible total score of 1–5

Further, we used multiple linear regression to explore associations between gender, HL, COVID-19-related worries and HRQoL in adolescents. KIDSCREEN-10 was the dependent variable. Assumptions for linear regression were checked and fulfilled. To estimate the associations between gender, HL, COVID-19-related worries and HRQoL in parents, we used robust regression analyses because the assumptions for multiple linear regression were not fulfilled. The two RAND-36 sum scores (PCS and MCS) were the dependent variables. Age and education level were entered as covariates. The results are presented as regression coefficients with 95% confidence intervals (CI). *P*-values ≤0.05 were considered statistically significant. All analyses were considered exploratory; hence, no correction for multiple testing was performed. All analyses were conducted using IBM SPSS Statistics (version 27)—except for the robust regression analyses, which were conducted using Stata (version 16).

## Results

### Descriptive sociodemographic characteristics of adolescents and parents

In total, 215 adolescents and 320 parents participated in the study (Table 1). Most were girls (69.3%) and women (81.0%), respectively. The median age for the adolescents was 16 years, and the mean (SD) age for the parents was 47.6 (4.6) years. Among the adolescents, most lived with both parents (71.6%), had parents who were both born in Norway (76.3%) and had parents who were both working (81.9%). Among the parents, most were married or cohabiting (78.8%), had higher education of 4 years or more (58.1%), worked full time (78.1%) and had a household income of more than one million NOK/year (58.8%).

### Descriptive data for HRQoL, HL and COVID-19-related worries of adolescents and parents

Table 2 shows the descriptive data for HRQoL, HL and COVID-19-related worries in adolescents and parents. The adolescents’ mean (SD) for KIDSCREEN-10 was 44.3 (7.8). Boys reported significantly higher levels of HRQoL compared to girls. The adolescents’ median (min, max) score for HL was 34 (20, 40), with no significant gender differences. Adolescents reported that the COVID-19 pandemic had changed their lives in both a positive and a negative way. More girls reported a positive change; however, the proportion of adolescents who reported a considerably negative change was higher than the proportion who reported a positive change. Further, adolescents reported a high degree of COVID-19-related worries, especially concerning worries about infecting others with COVID-19 and about family/friends becoming sick with COVID-19. Girls were significantly more worried about becoming sick, infecting others, family/friends becoming sick and about the economy in Norway compared to boys.

The parents’ median (min, max) score for RAND-36 was 54.7 (14.7, 67.5) for PCS and 54.4 (10.1, 67.3) for MCS. Men reported significantly higher MCS values compared to women. Parents’ mean (SD) scores for the five HL domains were 3.2 (0.5) for the domain “having sufficient information to manage my health,” 3.0 (0.5) for “actively managing my health,” 2.9 (0.5) for “appraisal of health information,” 4.9 (0.5) for “ability to find good health information” and, finally, 4.1 (0.5) for the domain “understand health information well enough to know what to do.” There were no statistically significant gender differences, considering the parents’ HL. Parents reported that the COVID-19 pandemic had changed their lives both positively and negatively; however, a higher proportion of parents reported a negative change compared to the proportion of parents who reported a positive change. Considering COVID-19-related worries, parents were mostly worried about infecting others with COVID-19 and about family/friends becoming sick. A significantly higher proportion of women compared to men reported having worries related to becoming sick and infecting others and worries about family/friends becoming sick with COVID-19. Still, a significantly higher proportion of women compared to men reported a positive change in life due to the pandemic. Details are provided in Table 2.

### Associations between gender, HL, COVID-19-related worries and HRQoL in adolescents and parents

Table 3 shows the results from the multiple linear regression analysis of gender, HL, COVID-19-related worries and HRQoL in adolescents. Being a girl was significantly associated with lower HRQoL compared to being a boy (B = − 3.77; 95% CI [− 5.95; − 1.06]). Higher HL was significantly positively associated with increased HRQoL. As the adolescents’ HL score increased by one point, their HRQoL score increased by 0.52 (95% CI [0.28; 0.76]) points. There were no statistically significant associations between worries about infecting others with COVID-19, worries about family/friends becoming sick with COVID-19 and HRQoL.

**Table 3:** Associations between gender, health literacy, COVID-19-related worries and health-related quality of life in adolescents (*N* = 215) ^a, b^

	B	95% CI	***P***-value
Gender (ref = boy)	−3.72	−5.89 – − 1.55	**.001**
Health literacy ^c^	0.53	0.29–0.77	**<.001**
Worried about infecting others with COVID-19 (ref = a little worried) ^d^			
Not worried at all	0.53	−4.07 – 5.14	.820
Worried a lot	−0.88	−3.34 – 1.59	.485
Worried about family/friends becoming sick with COVID-19 (ref = a little worried) ^d^			
Not worried at all	−0.71	−5.10 – 3.68	.751
Worried a lot	−2.23	−4.54 – 0.08	.059

B, unstandardized coefficient, *CI* Confidence interval

*P*-values marked with bold print indicate statistically significant differences between the groups (*P* ≤ 0.05)

^a^Multiple linear regression analysis

^b^Health-related quality of life was analyzed with KIDSCREEN-10. Higher values indicate higher levels of health-related quality of life

^c^Sum score obtained from the Health Literacy in School-Aged Children questionnaire. Higher scores indicate higher levels of health literacy

^d^The variable was recoded into three categories: “Not worried at all,” “A little worried” and “Worried a lot” (quite worried, worried a lot)

Table 4 shows the results from the robust regression analyses between gender, HL, COVID-19-related worries and HRQoL in parents. There was no statistically significant association between gender and PCS or gender and MCS. Higher HL in the domain “understand health information well enough to know what to do” was significantly associated with lower levels of HRQoL for PCS (B = − 2.68; 95% CI [− 4.64; − 0.72]) and higher levels of HRQoL for MCS (B = 4.62; 95% CI [1.72; 7.52]). There were no significant associations between the other four HL domains and PCS or MCS. For MCS, being worried a lot about infecting others was significantly associated with higher HRQoL compared to being a little worried (B = 2.69; 95% CI [0.52; 4.86]). Further, being worried a lot about family/friends becoming sick was significantly associated with lower HRQoL compared to being a little worried (B = − 3.84; 95% CI [− 5.97; − 1.72]). For PCS, there was no statistically significant association between COVID-19-related worries and HRQoL.

**Table 4:** Associations between gender, health literacy, COVID-19-related worries and health-related quality of life in parents (*N* = 320) ^a^

Physical component summary ^**b**^	B	95% CI	***P***-value
Gender (ref = man)	−0.11	− 1.56 – 1.34	.885
Health literacy			
Having sufficient information to manage my health ^c^	1.04	−0.62 – 2.71	.217
Actively managing my health ^c^	0.92	−0.40 – 2.25	.172
Appraisal of health information ^c^	−0.34	−1.63 – 0.94	.598
Ability to find good health information ^c^	1.56	−0.35 – 3.47	.110
Understand health information well enough to know what to do ^c^	−2.68	−4.64 – −0.72	**.007**
Worried about infecting others with COVID-19 (ref = a little worried) ^d^			
Not worried at all	0.11	−1.65 – 1.88	.898
Worried a lot	−0.68	−2.15 – 0.78	.357
Worried about family/friends becoming sick with COVID-19 (ref = a little worried) ^d^			
Not worried at all	1.36	−0.59 – 3.31	.172
Worried a lot	−0.28	−1.71 – 1.16	.705
**Mental component summary** ^**b**^	**B**	**95% CI**	***P*-value**
Gender (ref = man)	−1.17	−3.32 – 0.98	.285
Health literacy			
Having sufficient information to manage my health ^c^	2.10	−0.36 – 4.56	.095
Actively managing my health ^c^	0.67	−1.29 – 2.64	.501
Appraisal of health information ^c^	−1.41	−3.31 – 0.49	.145
Ability to find good health information ^c^	−2.08	−4.92 – 0.74	.148
Understand health information well enough to know what to do ^c^	4.62	1.72–7.52	**.002**
Worried about infecting others with COVID-19 (ref = a little worried) ^d^			
Not worried at all	−1.60	−4.21 – 1.02	.230
Worried a lot	2.69	0.52–4.86	**.015**
Worried about family/friends becoming sick with COVID-19 (ref = a little worried) ^d^			
Not worried at all	1.70	−1.19 – 4.59	.248
Worried a lot	−3.84	−5.97 – −1.72	**<.001**

B, unstandardized coefficient, *CI* Confidence interval

*P*-values marked with bold print indicate statistically significant differences between the groups (P ≤ 0.05)

^a^Robust regression analysis with control for age and education level

^b^RAND-36 sum scale. Higher values indicate higher levels of health-related quality of life

^c^Sum scores obtained from Health Literacy Questionnaire domains. Higher scores indicate higher levels of health literacy

^d^The variable was recoded into three categories: “Not worried at all,” “A little worried” and “Worried a lot” (quite worried, worried a lot)

## Discussion

One of the main findings of this study was that the adolescents’ HRQoL scores were notably lower compared to European norms [38] and the results of previous studies among Norwegian adolescents [50, 51]. This corresponds with recent studies among adolescents during the pandemic [4, 16, 20, 21], indicating that the uncertain and demanding situation caused by the pandemic has had a major negative impact on the physical, psychological, functional and social aspects of adolescents’ health. The result is unsurprising, considering the increase in mental health problems and the high prevalence of loneliness, stress, worries and uncertainty related to COVID-19 found in other studies during the initial phase of the pandemic [3, 4, 8, 15, 22].

In contrast, parents’ HRQoL scores were comparable to Norwegian normative data [27, 52]. Parents have also experienced great challenges caused by the pandemic—for example, changes in everyday routines, social restrictions, the use of home offices and possible financial challenges while having to take care of the family as well. Still, parents’ HRQoL scores seem to be rather stable. There is diversity in the literature related to adults’ HRQoL during the pandemic, which may be explained through differences related to being quarantined, SES, work situation, financial concerns and/or existing physical or mental health problems [10, 22, 23].

The adolescents reported moderate-to-high levels of HL [44]. Considering the association between HL and adolescents’ health behaviors [29], our findings indicate that Norwegian adolescents have the necessary skills to understand and act upon COVID-19-related information. This highlights the importance of including adolescents in communication about the pandemic and matters related to their health behaviors, as this will empower adolescents in health decision-making processes. Further, this study provides insight into the impact of HL on adolescents’ HRQoL, confirming the positive association between HL and HRQoL [21, 31]. Therefore, one would assume that the adolescents’ HRQoL scores were higher. However, high HL may have caused high fidelity considering COVID-19-restrictions, which according to Riiser et al. [21], requires great sacrifice because the pandemic strategies seem to conflict with aspects that are important for adolescents’ HRQoL (e.g., socializing with friends and participating in leisure activities). This might explain our results.

The high HL levels reported by the parents indicate higher HL levels compared to the results of a recent Norwegian national HL survey [53]. The parents’ HL scores for the five HLQ domains indicate they are up to date, feel confident they have all the necessary information, can identify and understand information and reliable sources of information and can make good decisions about health [25]. In contrast, the recent national survey found that a significant proportion of the Norwegian population finds it difficult to critically assess health information and experience that it is challenging to assess the advantages and disadvantages of different treatment options [26]. Our findings could possibly be explained by a high SES in our sample. Both Norwegian and international studies have shown that education affects HL, with higher education associated with higher HL levels [26–29]. Further, regarding the association between HL and HRQoL in parents, our results confirm that higher HL is associated with higher HRQoL [26, 33] but only for mental well-being (MCS). Surprisingly, for physical well-being (PCS), results show that higher HL is associated with decreased PCS. We have no explanation for this association between HL and PCS; we may only speculate that increased health knowledge and understanding may lead to increased awareness of one’s physical condition. Moreover, only the domain “understand health information well enough to know what to do” was significantly associated with MCS and PCS, indicating that during the pandemic, this HLQ domain might be more important for parents’ HRQoL than the other four domains.

The proportion of adolescents and parents who reported a negative change in life due to the pandemic was higher than the proportion who reported a positive change. This indicates that although the pandemic might lead to improvements in some areas of life for adolescents and parents [15, 22, 23, 34], 1 year into the pandemic, this is outweighed by deteriorations in other important areas of life.

We found no statistically significant association between COVID-19-related worries and HRQoL in adolescents. Still, the high degree of worries reported by the adolescents, especially concerning worries about infecting others and about family/friends becoming sick, is in line with findings from the initial phase of the pandemic [14–16] and emphasizes the pandemic’s impact on adolescents’ psychological health. Interestingly, compared to parents, a higher proportion of adolescents reported being worried a lot, indicating that adolescents have more COVID-19-related worries. However, in parents, we did find a significant association between COVID-19-related worries and HRQoL. The results show that being worried a lot about family/friends becoming sick has a negative impact on parents’ MCS. Further, and surprisingly, being worried a lot about infecting others was significantly associated with higher MCS.

Our results confirm that male gender is associated with higher HRQoL in adolescents and parents [4–6, 9, 12, 31]. Interestingly, in women, we found a larger variation in HRQOL scores for both PCS and MCS compared to in men. Considering HL, we found no significant gender differences in adolescents or parents, indicating HL is equally distributed across gender in this sample population. However, despite similar levels of HL, girls and women reported significantly more COVID-19-related worries compared to boys and men, respectively. These results might be explained through gender-related differences shown in Galasso et al. [54], who found that women are more likely to perceive COVID-19 as a serious health problem, agree with restraining public policy measures and comply with them.

Unlike international studies that emphasize the burden of financial worries due to the pandemic [5, 10, 13], we found that most adolescents and parents were not worried about work and/or the economy. This may be explained by the Norwegian welfare system, which provides schemes such as pensions and unemployment benefits, sickness and child benefits for the Norwegian population [55]. Further, an important agenda for the Norwegian policy response to the COVID-19 crisis is to mitigate the impact on the economy [56], and the Norwegian government introduced significant measures to secure jobs, help people and businesses and strengthen health services [57]. Our findings indicate that the Norwegian population might be less affected by the economic impact of the pandemic compared to other countries.

### Limitations

This is a cross-sectional study; thus, causal inference cannot be determined. Another limitation is the response rate of only 33.2% among adolescents and 57.0% among parents. Unfortunately, due to General Data Protection Regulation laws, we do not have information to assess whether the nonparticipants and participants differ in any respect. However, our results indicate that most participating adolescents and parents come from families with high SES and that few have an immigrant background. Thus, the study results may not be representative of the whole population of Norwegian adolescents and parents. Previous research among adolescents and parents shows that HRQoL, HL, physical and mental consequences of and worries related to the pandemic vary across sociodemographic groups [4, 14, 15, 23, 26, 58]. Furthermore, most participants in the present study were girls (69.3%) and women (81.0%). Hence, a selection bias may exist in our findings. Together, this must be taken into consideration when interpreting our results.

### Implications and future research

Overall, this study contributes to more knowledge of HRQoL of 16- to 17-year-old adolescents and parents, their HL and COVID-19-related worries about 1 year into the pandemic. Importantly, the study provides insight into the impact of gender, HL and COVID-19-related worries on adolescents’ and parents’ HRQoL. Given the uncertainty of pandemic development in the future, these insights provide valuable information for interventions aiming to increase the well-being of adolescents and parents. This knowledge is highly relevant for public health and health policy, indicating that efforts aimed at increasing people’s HL might indirectly affect their HRQoL as well and that gender-specific interventions or strategies could be beneficial.

In line with previous research [2, 4, 12, 22], this study supports calls for strategies to enhance adolescents’ HRQoL and mental health during and after the pandemic by, for example, increasing adolescents’ access to mental health services and providing clear and correct information to parents, teachers and health professionals on how to help adolescents cope with emotions, stress and problem solving related to the pandemic. Further, as highlighted in previous studies, we emphasize that it is important to be particularly attentive to vulnerable groups whose mental health and HRQoL might already be poor—such as families with low SES, adolescents and/or parents with mental health problems or chronic diseases and people with problems related to violence, substance abuse or mental illness in close relatives [2, 4, 12, 14, 15, 21, 22].

More in-depth research (e.g., qualitative data) is needed to further explore factors that characterize those who experience a positive change in life and those who experience a negative change due to the pandemic. Further, we encourage future studies to use longitudinal designs and include a higher percentage of male gender, participants with low SES and an immigrant background to explore our findings more thoroughly. Making a short information video and/or using communication platforms such as social media to provide oral information about a study, might be a promising way to increase study recruitment among adolescents and their parents.

## Conclusions

Our results indicate that the pandemic has had a major negative impact on adolescents’ HRQoL. Parents’ HRQoL remained unchanged and comparable to previous studies. Our study demonstrates that HL, gender and COVID-19-related worries are significantly associated with adolescents’ and parents’ HRQoL, indicating that efforts aimed at increasing their HL might indirectly affect their HRQoL as well and that gender-specific interventions or strategies could be beneficial. We highlight the need for strategies to enhance adolescents’ HRQoL and mental health during and after the pandemic.

## Supplementary Information

**Additional file 1.** Cronbach’s alpha values for instruments used in this study. Cronbach’s alpha values for KIDSCREEN-10, RAND-36, The Health Literacy in School-Aged Children Questionnaire, The Health Literacy Questionnaire.

## Data Availability

The datasets used and/or analyzed during the current study are not publicly available due to General Data Protection Regulation laws but are available from the corresponding author on reasonable request and with permission from the Norwegian Centre for Research Data.

## Abbreviations

CI: Confidence intervals
COVID-19: Coronavirus disease 2019
HL: Health literacy
HLQ: The Health Literacy Questionnaire
HLSAC: The Health Literacy in School-Aged Children questionnaire
HRQoL: Health-related quality of life
MCS: Mental component sum scores
SD: Standard deviation
SES: Socioeconomic status
PCS: Physical component sum scores
RAND-36: The 36-Item Medical Outcomes Study Short Form
WHO: The World Health Organization
