# Supplementary material for: Health-related quality of life, health literacy and COVID-19-related worries of 16- to 17-year-old adolescents and parents one year into the pandemic: a cross-sectional study
Source: BMC Public Health. 2022 Jul 9;22:1321. doi: 10.1186/s12889-022-13737-1 (PMC9271239; doi:10.1186/s12889-022-13737-1)
Supplement: Supplementary file 1 — Additional file 1. Cronbach’s alpha values for instruments used in this study. Cronbach’s alpha values for KIDSCREEN-10, RAND-36, The Health Literacy in School-Aged Children Questionnaire, The Health Literacy Questionnaire. [file 12889_2022_13737_MOESM1_ESM.docx]

**Additional file 1. Cronbach’s alpha values for instruments used in this study**

| Factors | Instruments | Number  of items | α ^ab^ | α ^ac^ |
| --- | --- | --- | --- | --- |
| HRQoL | KIDSCREEN-10 | 10 | .81 |  |
|  | RAND-36 |  |  |  |
|  | Mental health | 5 |  | .85 |
|  | Vitality | 4 |  | .88 |
|  | Bodily pain | 2 |  | .84 |
|  | General health | 5 |  | .84 |
|  | Social function | 2 |  | .86 |
|  | Physical function | 10 |  | .90 |
|  | Role limitation (physical) | 4 |  | .89 |
|  | Role limitation (emotional) | 3 |  | .87 |
| HL | Health Literacy in School-Aged Children  The Health Literacy Questionnaire | 10 | .86 |  |
|  | Having sufficient information to manage my health | 4 |  | .81 |
|  | Actively managing my health  Appraisal of health information  Ability to find good health information  Understanding health information well enough to know what to do | 4  5  5  5 |  | .87  .75  .87  .81 |

ª Cronbach’s alpha coefficient values in this study, ^b^ in adolescents, ^c^ in parents

HRQoL, health-related quality of life; HL, health literacy
